# Supplementary material for: The Role of Mu Opioid Receptors in High Fat Diet-Induced Reward and Potentiation of the Rewarding Effect of Oxycodone
Source: Life (Basel). 2023 Feb 23;13(3):619. doi: 10.3390/life13030619 (PMC10055773; doi:10.3390/life13030619)
Supplement: Supplementary file 1 [file life-13-00619-s001.zip › life-2188601-supplementary.pdf]

**Supplemental Data:**

**Comparison of the amount of time that male and female C57BL/6J mice spent in the paired chamber (PCh) with that of MOR wildtype (MOR+/+) mice.** Figure S1 shows the amount of time that male and female MOR wildtype and C57BL/6J mice spent in the PCh. A mixed effect ANOVA revealed no significant effect of any factor or interaction between the factors except there was a significant effect of session ($P<0.001$). The detail of the data analysis is provided below. Thus, we combined the amount of time that mice of each sex spent in the PCh to have more power given that our study is underpowered (n= 6-8 mice of each sex per genotype).

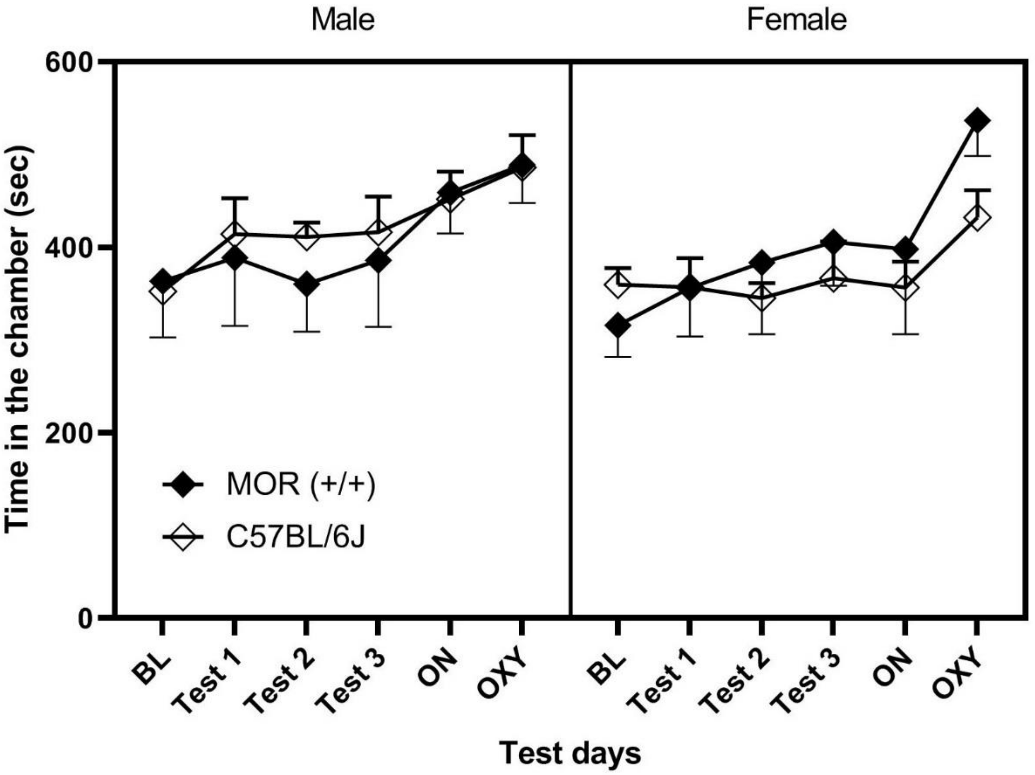


**Figure S1.** Comparison of the amount of time that male and female C57BL/6J mice spent in the paired chamber (PCh) in comparison with MOR wildtype mice. Data represent as mean ± standard error of the mean (± S.E.M) of the amount of time that mice spent in the PCh in male and female C57BL/6J (closed circles, n = 6 mice of each sex) and MOR (+/+) mice (n = 6-8 mice of each sex).

**Mixed ANOVA of the data in male C57BL/6J male HFD vs. RCD group**

| | | | |
|---|---|---|---|
| Table Analyzed | PCh in male and female MOR mice | | |
| | | | |
| Mixed-effects model (REML) | Matching by factor: Genotype | | |
| Assume sphericity? | Yes | | |
| Alpha | 0.05 | | |
| | | | |
| Fixed effects (type III) | P value | P value summary | F (DFn, DFd) |
| Session | 0.0007 | *** | F (5, 132) = 4.548 |
| Sex | 0.0994 | ns | F (1, 132) = 2.754 |
| Genotype | 0.6720 | ns | F (1, 132) = 0.1800 |
| Session x Sex | 0.8730 | ns | F (5, 132) = 0.3630 |
| Session x Genotype | 0.8774 | ns | F (5, 132) = 0.3564 |
| Sex x Genotype | 0.2319 | ns | F (1, 132) = 1.442 |
| Session x Sex x Genotype | 0.8454 | ns | F (5, 132) = 0.4039 |
| | | | |
| Random effects | SD | Variance | |
| Subject | 0.000 | 0.000 | |
| Residual | 113.7 | 12933 | |
| | | | |
| Was the matching effective? | | | |
| Chi-square, df | | | |
| P value | | | |
| P value summary | | | |
| Is there significant matching (P < 0.05)? | No | | |
| | | | |
| Data summary | | | |
| Number of columns | 2 x 2 | | |
| Number of rows (Session) | 6 | | |
| Number of subjects (Subject) | 84 | | |
| Number of missing values | 12 | | |
